# Discovery and Confirmation of O-GlcNAcylated Proteins in Rat Liver Mitochondria by Combination of Mass Spectrometry and Immunological Methods

**DOI:** 10.1371/journal.pone.0076399

**Published:** 2013-10-02

**Authors:** Weiqian Cao, Jing Cao, Jiangming Huang, Jun Yao, Guoquan Yan, Haoqi Xu, Pengyuan Yang

**Affiliations:** Institutes of Biomedical Sciences and Department of Chemistry, Fudan University, Shanghai, China; Swiss Institute of Bioinformatics, Switzerland

## Abstract

O-linked β-N-acetylglucosamine (O-GlcNAc) is an important post-translational modification (PTM) consisting of a single N-acetylglucosamine moiety attached via an O-β-glycosidic linkage to serine and threonine residues. Glycosylation with O-GlcNAc occurs on myriad nuclear and cytosolic proteins from almost all functional classes. However, with respect to O-GlcNAcylated proteins special in mitochondria, little attention has been paid. In this study, we combined mass spectrometry and immunological methods to perform global exploration of O-GlcNAcylated proteins specific in mitochondria of rat liver. First, highly purified mitochondrial proteins were obviously shown to be O-GlcNAcylated by immunoblot profiling. Then, β-elimination followed by Michael Addition with Dithiothreitol (BEMAD) treatment and LC-MS/MS were performed to enrich and identify O-GlcNAcylated mitochondrial proteins, resulting in an unambiguous assignment of 14 O-GlcNAcylation sites, mapping to 11 O-GlcNAcylated proteins. Furthermore, the identified O-GlcNAcylated mitochondrial proteins were fully validated by both electron transfer dissociation tandem mass spectrometry (ETD/MS/MS) and western blot. Thus, for the first time, our study definitely not only identified but also validated that some mitochondrial proteins in rat liver are O-GlcNAcylated. Interestingly, all of these O-GlcNAcylated mitochondrial proteins are enzymes, the majority of which are involved in a wide variety of biological processes, such as urea cycle, tricarboxylic acid cycle and lipid metabolism, indicating a role for protein O-GlcNAcylation in mitochondrial function.

## Introduction

O-linked β-N-acetylglucosamine (O-GlcNAc) is a ubiquitous modification with a single N-acetylglucosamine attachment to hydroxyl groups of Ser and/or Thr residues of target proteins, which occurs in all metazoas. O-GlcNAcylation is a nutrient and stress sensor involved in wide biological processes [1], such as transcription, cell structure, metabolism and cancer cell biology [2-5]. Unlike N-glycosylation or mucin-type O-glycosylation, O-GlcNAc is not elongated or further modified [4]. Cycling of O-GlcNAc is tightly regulated by the cooperation of two highly conserved enzymes O-GlcNAc transferase (OGT) and O-GlcNAcase. So far, thousands of proteins have been identified to be O-GlcNAcylated [1,6–11]. Almost all of these proteins are restricted to nucleus and cytosol [12]. Recently, a few studies have demonstrated that O-GlcNAcylation is also involved in other organelles, such as mitochondria [11,13-15].

Mitochondria are double-membrane organelles found in most eukaryotic cells [16]. They are the power centers of cells and are involved in a range of other processes [17,18]. Tyrosine kinases and phosphatases have been identified in mitochondrial compartments. Abundant functional mitochondrial proteins are demonstrated to be phosphorylated [19]. In many ways, O-GlcNAcylation is similar to O-phosphorylation: for instance, like phosphate, N-acetylglucosamine moiety can be attached and removed rapidly in response to internal or environmental changes [4,20,21]; and both O-GlcNAcylation and O-phosphorylation occur on Ser and/or Thr residues, which hints O-GlcNAcylation has a direct competition with O-phosphorylation [1]. Furthermore recent studies have revealed that besides phosphorylation on serine/threonine, also about 68.02% of the O-GlcNAcylated proteins are known to be tyrosine phosphorylated [22-24]. Thus, an increasing number of phosphorylated proteins have been found in mitochondria, and the site-specific interplay between O-phosphorylation and O-GlcNAcylation has been widely recognized.

In contrast to studies in nuclear and cytosolic O-GlcNAcylated proteins [25-29], O-GlcNAcylation of mitochondrial proteins has been explored in a limited form [13]. The identification of OGT isoform (mOGT) [30-32] which was preferred to be located in mitochondria suggested that O-GlcNAcylation could happen on mitochondrial proteins. In recent reports, several mitochondrial proteins have been identified to be O-GlcNAcylated [13,14]. For example, altered O-GlcNAc modification and phosphorylation of mitochondrial proteins have been investigated in myoblast cells exposed to high glucose [14]. However, the definite proteins/peptides and exact amino acids sites with O-GlcNAc modification were seldom known [14], because the evidence of mitochondrial protein O-GlcNAcylation in these studies was almost got from immunological methods, such as immunoprecipitation and western blot with antibody RL2 or lectin WGA. To our knowledge, in the reports produced by mass spectrometry (MS) [11,13], a few O-GlcNAcylated proteins probably came from mitochondria. However, few of the MS results were validated by other independent methods and the identified O-GlcNAcylated proteins may come from other organelles but mitochondria, because no purified mitochondrial samples were used. In fact, O-GlcNAcylation in mitochondrial protein is largely unexplored in particular. Thus, we are motivated to not only discover but also validate O-GlcNAcylated proteins specific in mitochondria of rat liver definitely.

As with any PTM, site mapping is a prerequisite toward understanding the biological function of the modifications. Mapping O-GlcNAc attachment sites is very difficult due to its chemical labile and low stoichiometry at any site on a protein. To compensate for the substoichiometric occupancy of O-GlcNAc modification, numerous methods have been developed for enrichment and detection, such as immunoaffinity/lectin chromatography [33,34], tagging-via-substrate (TAS) method [35], chemoenzymatic approach [36] and β-elimination/Michael Addition-based enrichment method [37]. Immunoaffinity/lectin chromatography is the simplest approach to purify O-GlcNAcylated proteins. However, this method prefers to enrich high abundance proteins or those with multiple clustered O-GlcNAc residues instead of low abundance proteins with single or widely separated O-GlcNAcylation sites [1]. The TAS approach is useful for studying abundant O-GlcNAcylated proteins in living cells [34,38]. Chemoenzymatic approach [39], although can greatly enrich O-GlcNAcylated proteins/peptides and can be combined with ETD/MS/MS analysis for sites mapping, it is still not suitable for sites mapping for high-through put by direct CID/CAD MS/MS for its labile and large mass of tag attached to the O-GlcNAc [1]. Recently, photochemical cleavage approach had been developed to help in O-GlcNAc site mapping in combination with chemoenzymatic [40]. β-elimination followed by Michael Addition at O-GlcNAcylated serine or threonine with DTT (BEMAD) for enrichment and sites mapping has been employed to facilitate mapping of O-GlcNAcylation sites. In addition to enabling enrichment of low abundant modified peptides using thiol-afﬁnity chromatography, the attached DTT moiety is stable for collision-induced dissociation (CID) [41,42].

Thus, in our study, we took advantage of BEMAD method and combined tandem mass spectrometry and immunological methods to identify and validate the O-GlcNAcylated proteins in rat liver mitochondria, and tried to explore the role of such modification in mitochondria.

## Materials and Methods

### 2.1: Chemicals and reagents

Nycodenz was purchased from Axis-shield (Oslo, Norway). Sequencing grade trypsin and alkaline phosphatase were obtained from Promega (Madison, WI, USA). Complete protease inhibitor mixture tablets were purchased from Roche Applied Science (Basel, Switzerland). Thiopropyl Sepharose 6B was purchased from Amersham Biosciences (Piscataway, NJ, USA). Sep-Pak C18 columns were purchased from Waters (Milford, MA, USA). All other chemicals were purchased from Sigma-Aldrich (St. Louis, MO, USA). Antibodies against the following proteins were used: COX3, GAPDH, c-Jun, RL2, CTD110.6, goat anti-rat IgG-CFL 594 (Santa Cruz, California, USA); ATP synthase subunit beta (ATPB), Cytochrome P450 1A1+1A2 (CP1A1+1A2) (Abcam, Cambridge, UK); Long-chain-fatty-acid--CoA ligase 1 (ACSL1) (Cell Signaling Technology, Beverly, MA, USA). ECL plus detection system was obtained from GE Healthcare (Piscataway, NJ, USA). Water used for all experiments was produced by a Milli-Q Plus system from Millipore (Bedford, MA, USA).

### 2.2: Rats

Rat liver tissues were obtained from healthy adult SD rats (270±30 g). The rats were obtained from the Science Department of Experimental Animals of Fudan University in China. All rats were housed with pathogen-free food and water under 12 h light-cycle conditions. The study was approved by the Review Board of Fudan Shanghai Medical College. All surgery was performed under chloral hydrate anesthesia, and all efforts were made to minimize suffering.

### 2.3: Mitochondria preparation

Livers from 6 healthy adult rats were mixed on equal weight. Mitochondria were isolated using the method previously described [43,44] with minor modification. Briefly, liver mixture was rinsed with ice-cold PBS buffer and diced into several small pieces. The tissue in 5 volumes of homogenization medium (250 mM Sucrose, 5 mM MgCl_2_, 0.2 mM Na _3_VO_4_, 1 mM NaF, protease inhibitor cocktail and 50 mM Tris-HCl, pH 7.4) was homogenized in a glass homogenizer (Dounce loose-fitting). After being filtered through a 110-mesh filter, the homogenate was centrifuged (1000 g) three times at 4 °C for 10 min to remove unbroken cells and nucleus, and then centrifuged (15000 g) at 4 °C for 30 min. The supernatant was discarded and the pellets were washed in ice-cold washing buffer (200 mM Mannitol, 50 mM Sucrose, 1 mM EDTA, 0.5 mM EGTA, 0.2 mM Na _3_VO_4_, 1 mM NaF and 10 mM Tris-HCl, pH 7.4) for two times and then used for mitochondria purification through Nycodenz density gradient centrifugation.

The purified mitochondrial pellets from Nycodenz density gradient centrifugation were suspended in lysis buffer (8 M urea, 2 M thiourea, 1 mM PMSF and protease inhibitor cocktail) and NETN buffer (50 mM Tris pH 8, 100 mM NaCl, 1 mM EDTA, 0.5% NP40) respectively. Then the suspended proteins were sonicated three times at 4 °C, 100 W, for 5 s. After centrifugation (25000 g) at 4 °C for 45 min, the supernatant was collected and stored at -80 °C. The protein concentration was determined by the Bradford method.

### 2.4: Western blot analysis

The mitochondrial proteins in lysis buffer were separated by SDS-PAGE and transferred to PVDF membranes. The membranes were blocked in 5% nonfat milk. Antibodies against following proteins were used to demonstrate the purity of isolated mitochondria: COX3 (1:5000), GAPDH (1:2500), c-Jun (1:5000). RL2 antibody (1:1000) was used to detect O-GlcNAc modification. After incubation with respective antibodies, the membranes were washed with TBST (TBS with 0.1% Tween-20) and incubated in a 1:8000 dilution of HRP-conjugated IgG for 50 min at room temperature. Then, the membranes were washed with TBST and visualized using an ECL plus detection system. Before the membrane was incubated with RL2, it was blotted with RL2 plus 1 M GlcNAc to exclude nonspecific binding of the RL2 antibody.

### 2.5: Tryptic digestion of samples and phosphatase treatment

The proteins were in-solution digested using the method previously described [45] with minor modification. The mitochondrial proteins were reduced by 10 mM dithiothreitol (DTT) at 37 °C, alkylated by 20 mM iodoacetamide (IAA) in dark, at room temperature for 30 min. After IAA deactivation, the sample solution was diluted 10-fold with 50 mM NH_4_HCO_3_ buffer. Trypsin was added to the sample (1:50, w:w), incubated overnight at 37 °C. All digested peptide mixture was passed over a C18 column to remove extra DTT and salt. The peptides were eluted from the column with 30% acetonitrile (ACN) in 0.1% trifluoroacetic acid (TFA), followed by 70% ACN in 0.1% TFA. The peptide solution was dried in a vacuum centrifuge.

The digested peptides were suspended in 50 mM NH_4_HCO_3_ buffer and subjected to dephosphorylation with alkaline phosphatase (1 unit/10 μL) at 37 °C for 12 h. After incubation, TFA was added to stop the reaction and peptide mixture was dried in a vacuum centrifuge for later use.

### 2.6: Enrichment of O-GlcNAcylated peptide by β-Elimination/Michael Addition with DTT (BEMAD)

O-GlcNAcylated peptides were enriched using the method previously described [46] with a little modification. The peptides were suspended in the solution (pH 12-pH 13) containing 1.5% triethylamine, 0.15% NaOH, and 20 mM DTT, and incubated at 56 °C for 1.5 h. The reaction was immediately quenched by the addition of TFA to 2%. The peptides were desalted and dried for thiol enrichment. Thiolsepharose 6B was swelled in the TBS-EDTA (20 mM Tris, pH 7.6, 150 mM NaCl, 1 mM EDTA). The slurry was transferred to a microcentrifuge tube and washed 7 times with TBS-EDTA. The peptides were suspended in TBS-EDTA and mixed with thiolsepharose for 3 h at room temperature, then washed 7 times with TBS-EDTA and incubated with 20 mM DTT in TBS-EDTA for 1 h at room temperature. After incubation, the supernatant was collected, desalted and dried thoroughly for MS analysis.

### 2.7: Automated Nano-LC-ESI-MS/MS analysis of peptides

The peptides were suspended in 5% ACN in 0.1% formic acid (phase A), separated by nano-LC and analyzed by online electrospray tandem mass spectrometry in positive mode. Analysis of peptides were carried out by an LC-20AB system (Shimadzu, Tokyo, Japan) connected to an LTQ Orbitrap mass spectrometer (Thermo Electron, Bremen, Germany) equipped with an online nano-electrospray ion source (Michrom Bioresources, Auburn, CA). The separation of peptides took place in a 15-cm reverse phase column (100 µm i.d., Michrom Bioresources, Inc., Auburn, CA).

The peptide mixture was injected onto the trap-column with a flow rate of 20 µL/min and subsequently eluted with a gradient of 5% to 45% phase B (95% ACN in 0.1% formic acid) over 130 min, and then injected into the mass-spectrometer at a constant column-tip flow rate of 500 nL/min. The electrospray voltage was 1.6 kV. Eluted peptides were analyzed by MS and data-dependent MS/MS acquisition, selecting the 8 most abundant precursor ions for MS/MS with dynamic exclusion duration of 60 s. The scan range was set from m/z 350 to m/z 1800.

### 2.8: Data exploration

Data from ESI-MS/MS analysis was searched against Swiss-Prot database (Swissprot rat version 090303 with 7302 entries) by SEQUEST. Parameters were set as follows: enzyme, trypsin (partially enzymatic); maximum missed cleavages (MCs), two; variable modifications, oxidation (M, +15.99), carboxyamidomethylation (C, +57.052), alkylated cysteines became derivatized using DTT (C, +120.2), O-GlcNAc (S/T, +136.2); peptide tolerance, 10 ppm; and fragment tolerance, 1 Da. Trans-Proteomic Pipeline (TPP) [47] was used to further validate results obtained from SEQUEST. Database search results were statistically analyzed using PeptideProphet [48]. A minimum PeptideProphet probability score (P) filter of 0.9 was selected to remove the results with low-probability results. Here only those peptides passed the peptide probability threshold 0.9 and proteins passed the protein probability threshold 0.95 were accepted for further data interpretation.

### 2.9: Immunological validation

The mitochondrial proteins in NETN buffer were incubated with protein G-coupled sepharose beads for 30 min at 4°C with gentle agitation to remove non-specific binding proteins. After centrifugation (14000 g) at 4 °C for 10 min, supernatant was collected for immunoprecipitation. On the ice, 1 mg mitochondrial proteins were added in a tube plus antibodies (ATPB, CP1A1+1A2, ACSL1) at 1 µg/mL respectively, and incubated at 4 °C overnight with agitation. The protein G-coupled sepharose beads were rinsed twice with PBS, and mixed with 1% BSA/PBS (w/v) for 1 h. After that, the beads were washed with ENT buffer (50 mM Tris-HCl pH 8, 100 mM NaCl, 1 mM EDTA) twice. Then the beads were added to the sample. The lysate-beads mixture was incubated at 4 °C under rotary agitation for 4 hours. After incubation, the supernatant was removed and the beads were washed with ENT buffer three times. Finally, 2 × sample buffer (100 mM Tris-HCl pH 6.8, 4% SDS, 20% glycerol, 0.2 M DTT) was added into the beads, and incubated for 2 hours to denature the protein. Then the proteins in supernatant were separated from beads, collected, and analyzed by SDS-PAGE and western blot.

The proteins obtained from immunoprecipitation were run on 10% SDS-PAGE, then stained by silver staining or transferred to PVDF membranes. The membranes were immunoblotted with RL2 and CTD110.6 to detect the O-GlcNAc, and with goat anti-rat IgG-CFL 594 to exclude the IgG contamination as described above respectively. The same membranes were stripped using stripping buffer and reblotted with specific antibodies against the immunoprecipitated proteins.

### 2.10: ETD/MS/MS validation

Nano-LC-ESI-ETD/MS/MS was performed to validate the identified O-GlcNAcylated peptides. Briefly, the tryptic peptides from mitochondria were suspended in 5% ACN in 0.1% formic acid (phase A), separated by a 50-cm reverse phase column (75 µm i.d., Thermo, Fisher Scientific, Bremen, Germany) with a gradient of 5%-90% phase B (95% ACN with 0.1% formic acid) over 240 min, and analyzed by Orbitrap Elite ETD (Thermo Electron, Bremen, Germany) equipped with an online nano-electrospray ion source (Michrom Bioresources, Auburn, CA). The electrospray voltage was used at 2.5 kV. The peptides were analyzed by directed mass spectrometry with the identified O-GlcNAcylated peptides list. The scan range was set from m/z 400 to m/z 1800. The acquired MS/MS spectra were interpreted manually combining with Proteome Discoverer 1.4 software [49].

### 2.11: Bioinformatics analysis

Ingenuity Pathways Analysis (IPA, Ingenuity Systems, Mountain View, CA) was employed to assign the identified O-GlcNAcylated mitochondrial proteins into pathways and sub networks. Hypothetical protein interaction clusters were obtained through an updated “Ingenuity Pathways Knowledge Base (IPKB)” based on the findings of biological information on interactions between genes, proteins and other biological molecules. A data set containing the Swiss-Prot accession number of all the identified O-GlcNAcylated mitochondrial proteins was uploaded into the IPA server. The connectivity networks of the related candidate proteins were extracted by IPA. The network generated by ranking scores was optimized to include as many inputted protein as possible and to maximize networks connections. Meanwhile, the global canonical pathways that are significantly associated with these candidates were generated through IPA.

The protein subcellular location was got from the UniProtKB resources (http://www.uniprot.org). The phosphorylation status of the identified O-GlcNAcylated mitochondrial proteins was analyzed at PhosphoSitePlus (http://www.phpsphosite.org).

**Table 1:** A list of the identified O-GlcNAcylation sites of mitochondrial proteins.

SWISS-PROT accession no.	Protein description	gene name	Peptide sequence	Site
P07756	Carbamoyl-phosphate synthase	Cps1	MAS#TGEVACFGEGIHTAFLK	1331
P07756	Carbamoyl-phosphate synthase	Cps1	MAST#GEVACFGEGIHTAFLK	1332
P07756	Carbamoyl-phosphate synthase	Cps1	VLGTS#VESIMATEDR	537
P29147	D-beta-hydroxybutyrate dehydrogenase	Bdh1	FGVEAFS#DCLR	219
P04636	Malate dehydrogenase	Mdh2	VAVLGAS#GGIGQPLSLLLK	33
P10719	ATP synthase subunit beta	atp5B	LVLEVAQHLGES#TVR	106
P15999	ATP synthase subunit alpha	atp5A1	VLS#IGDGIAR	76
P24329	Thiosulfate sulfurtransferase	Tst	VLDAS#WYSPGTR	35
P10688	1-phosphatidylinositol-4,5-bisphosphate phosphodiesterase delta-1	Plcd1	KIFRECDHS#QTD	191
P10688	1-phosphatidylinositol-4,5-bisphosphate phosphodiesterase delta-1	Plcd1	KIFRECDHSQT#D	193
P18163	Long-chain-fatty-acid--CoA ligase 1	Acsl1	QVAEMAECIGS#ALIQK	136
P12939	Cytochrome P450 2D10	Cyp2d10	ITS#CDIEVQDFVIPK	382
P04799	Cytochrome P450 1A2	Cyp1a2	LS#QQYGDVLQIR	68
P00185	Cytochrome P450 1A1	Cyp1a1	LS#QQYGDVLQIR	71

## Results and Discussion

### 3.1: High purity and integrity of mitochondrial fractions from rat liver

**Figure 1:**
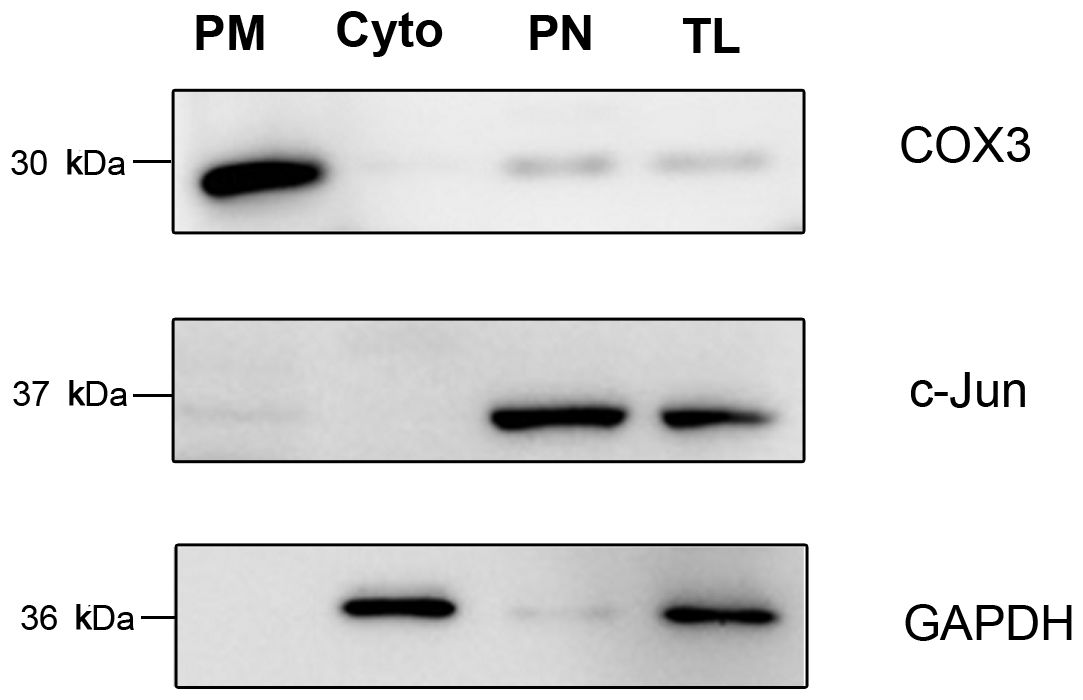
Purity and integrity of mitochondrial fractions. Mitochondrial proteins were applied to western blot. Cytosolic proteins, nuclear proteins and total rat liver proteins were also applied to western blot. Mitochondria marker COX3, nucleus marker c-Jun, and cytosol marker GAPDH were used to detect the purity and integrity of mitochondria. PM: purified mitochondria; Cyto: cytosol; PN: purified nucleus; TL: total liver.

Mitochondria were isolated from 6 adult rat livers using the method which has been proven and repeatedly cited [43,44]. Cytosol proteins and nuclear proteins were also collected as control. The purity and integrity of mitochondria were validated by western blot (Figure 1). Purity of mitochondria was excellent as visualized by organelle-specific markers. As shown in Figure 1, mitochondria marker COX3 showed significantly enrichment in mitochondrial fraction, while cytosol marker GAPDH and nucleus marker c-Jun were distinctly absent, indicating the purity of isolated mitochondria. In addition, COX3 was only present in mitochondrial fraction but not in cytosol and nucleus fractions, demonstrating the integrity of the mitochondrial extract. The high purity and integrity of the mitochondria ensured the fully exploration of O-GlcNAcylation specific in mitochondria instead of in other cell organelles.

### 3.2: Exploration of O-GlcNAcylation of mitochondrial proteins

#### 3.2.1: Assessment of O-GlcNAcylation of mitochondrial proteins by western blot

**Figure 2:**
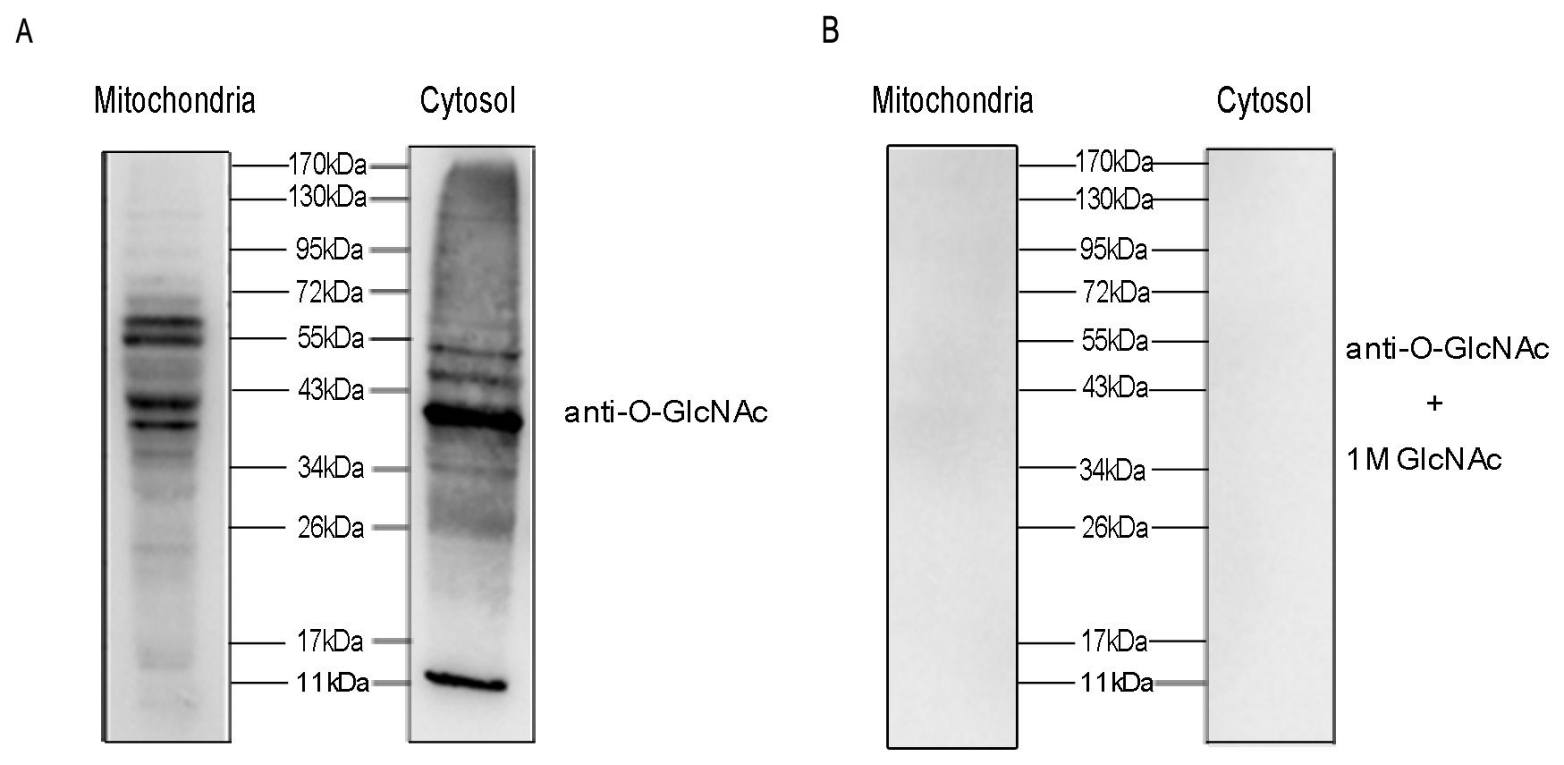
The level of O-GlcNAcylation in mitochondrial and cytosolic fractions. Mitochondrial and cytosolic proteins from rat livers were separated and analyzed by western blot. The membrane was probed with RL2 (A), and RL2 plus 1 M GlcNAc to exclude nonspecific binding of RL2 antibody (B).

To determine whether O-GlcNAcylation occurs on mitochondrial proteins, 20 µg pure mitochondrial proteins and cytosolic proteins isolated from rat livers were separated by SDS-PAGE and submitted to western blot with RL2 antibody (Figure 2). As shown in Figure 2A, O-GlcNAc is observed on mitochondrial proteins of several gel bands. Although the level of O-GlcNAcylation of mitochondrial proteins are less than that of cytosolic proteins, there are some marked O-GlcNAcylated mitochondrial proteins, especially the protein band between 34 kDa to 72 kDa. The immunoreactivity of the proteins with antibody RL2 is specific because 1 M GlcNAc competed away the signal (Figure 2B). Moreover, the purity and integrity of mitochondria ensure that the O-GlcNAc-modified proteins are derived from mitochondria, not from contamination of proteins from the nucleus or cytosol fractions.

#### 3.2.2: Mapping of O-GlcNAcylation sites on mitochondrial proteins with tandem mass spectrometry

Since we have found the existence of O-GlcNAc in multiple mitochondrial proteins by western blot, we further explored what these O-GlcNAcylated proteins were and where the O-GlcNAcylation sites located by mass spectrometry. Because O-GlcNAc is substoichiometric and labile, it is particularly difficult to be detected by conventional mass spectrometry techniques. BEMAD treatment can β-eliminate O-GlcNAc and replace it with a more stable DTT tag, which can be discernible by database searching. Mapping of DTT-modified sites assign the original O-GlcNAc modification sites on proteins. Thus, tryptic peptide mixture from isolated mitochondrial proteins were treated with BEMAD, after that, DTT-labeled peptides were purified by thiol column and finally detected by LC-MS/MS. Three biological replicates were performed and very strict database searching conditions were used in this study, resulting in an assignment of 14 O-GlcNAcylation sites on 12 O-GlcNAcylated peptides, mapping to 11 O-GlcNAcylated proteins (Table 1).

**Figure 3:**
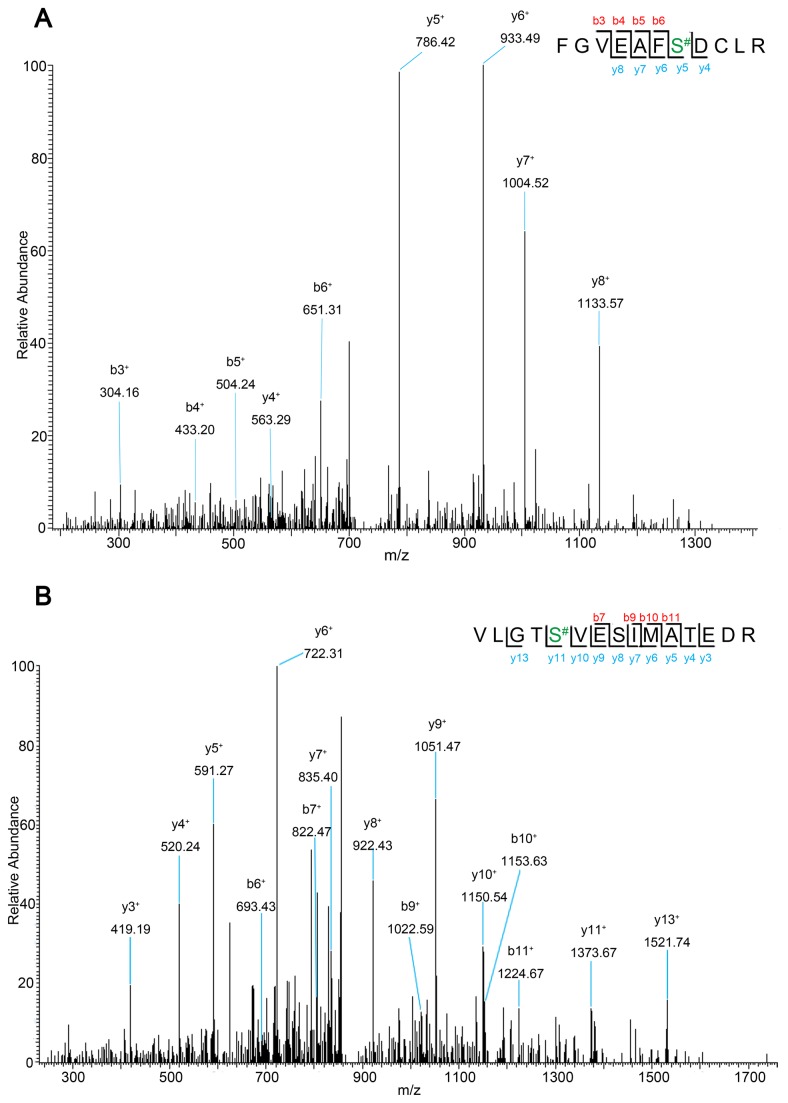
Nano-LC-MS/MS mass spectra of doubly charged O-GlcNAcylated peptides. FGVEAFS#DCLR [(M+2H)^2+^ at m/z 718.87] (A) from D-beta- hydroxybutyrate dehydrogenase, and VLGTS#VESIMATEDR [(M+2H)^2+^ at m/z 872.45] (B) from carbamoyl-phosphate synthase.

Figure 3A shows a MS/MS spectra of an identified O-GlcNAc-modified peptide [(M +2H) ^2+^ at m/z 718.87] from D-beta-hydroxybutyrate dehydrogenase (Bdh1) as an example. The location of the O-GlcNAcylation site (Ser-219) is determined by a differential mass of 136.2 Da to Ser after O-GlcNAc replaced by DTT. The y- series of product ions clearly display a mass shift indicating a DTT addition. Figure 3B depicts the location of an O-GlcNAcylation site (Ser-537) on the identified peptide [(M +2H)^2+^ at m/z 872.45] from carbamoyl-phosphate synthase (Cps1); peptide fragment present a mass of 223 Da on Ser-537, because of DTT addition as a sign for the O-GlcNAc modification site. In this study, the possibility of β-elimination/Michael addition to phosphorylated serine and threonine was almost ruled out by the fact that tryptic peptides were dephosphorylated with alkaline phosphatase before they were subjected to BEMAD treatment.

### 3.3: Verification of identified O-GlcNAcylated mitochondrial proteins

**Figure 4:**
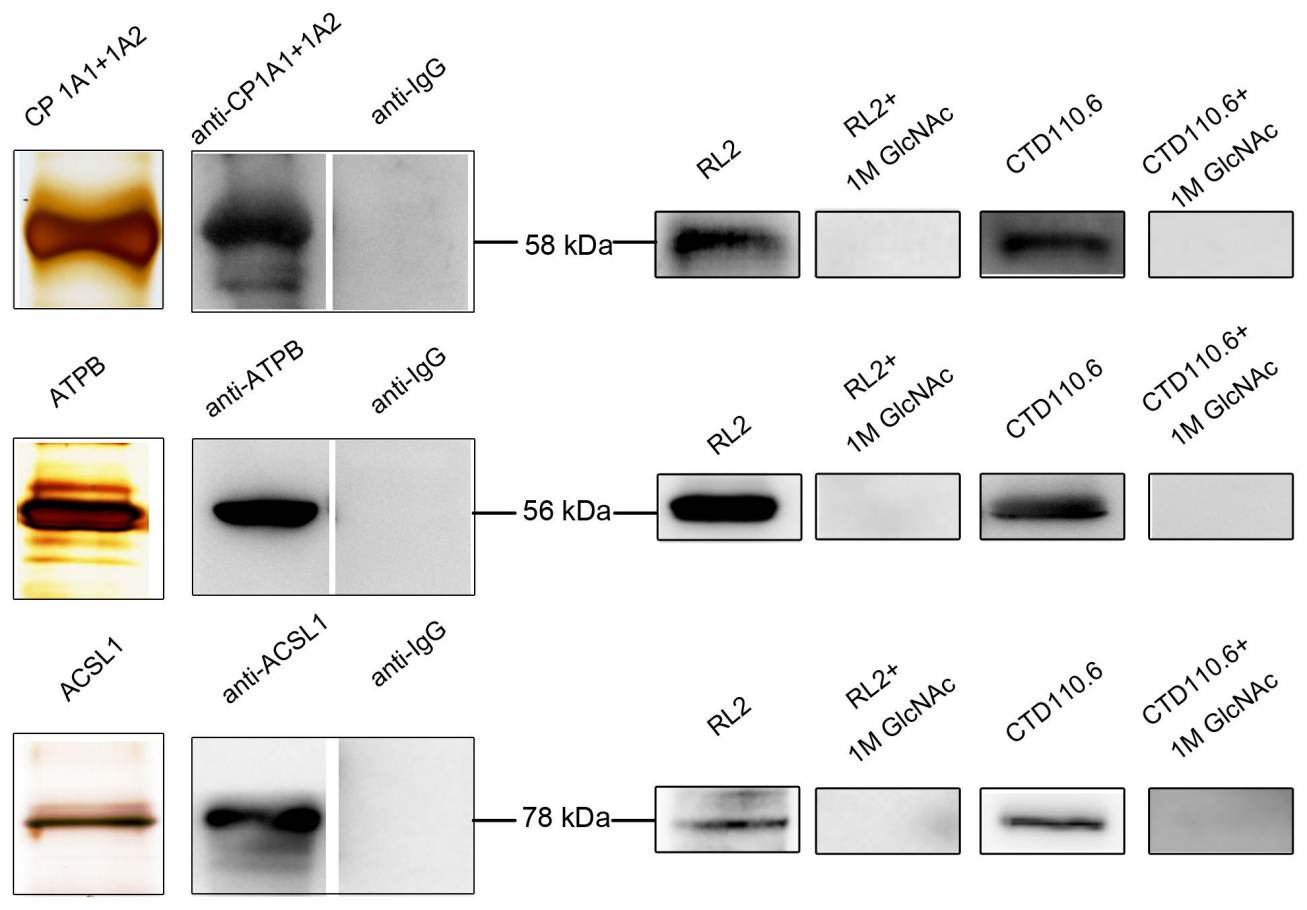
Verification of O-GlcNAcylated mitochondrial proteins. First, three O-GlcNAcylated mitochondrial proteins, cytochrome P450 (CP1A1+1A2), ATP synthase subunit beta (ATPB) and Long-chain-fatty-acid--CoA ligase 1 (ACSL1), were immunoprecipitated from rat liver mitochondrial proteins respectively. Then, the purified proteins were analyzed by silver staining and western blot with the specific antibodies. Stringently, goat anti-rat IgG-CFL 594 was also used to exclude non-specificity. Furthermore, the proteins were detected with RL2 and CTD110.6 to confirm their O-GlcNAcylation. Strictly, the competition experiments with 1 M GlcNAc was performed to exclude nonspecific binding of RL2 and CTD110.6 antibody.

To validate these identified O-GlcNAcylated mitochondrial proteins, ATP synthase subunit beta, cytochrome P450, Long-chain-fatty-acid--CoA ligase 1 proteins, of which antibodies are commercially available, were immunoprecipitated from rat liver mitochondrial proteins respectively. Then, the immunoprecipitated proteins were separated by SDS-PAGE, followed by silver staining and immunoblot with O-GlcNAc antibody RL2, CTD110.6 and goat anti-rat IgG-CFL 594 respectively. The same membranes were stripped and reblotted with the specific antibodies to confirm the identity of the proteins. As shown in Figure 4, the combined results of silver staining and immunoblot with the specific antibodies demonstrate that all of these proteins are successfully isolated from the complex sample. Besides, more strictly for the immunoblot positive results, the negative results of anti-IgG as control exclude the possibility of contamination from IgG heavy chain clearly. The proteins detected by RL2 and CTD110.6 showing a significant signal indicate that these proteins are shown to be O-GlcNAcylated. Competition experiments with 1 M GlcNAc exclude nonspecific binding of the RL2 and CTD110.6 antibody (Figure 4). So, by these specific and exclusive steps, these proteins are proven to be O-GlcNAcylated unambiguously.

**Figure 5:**
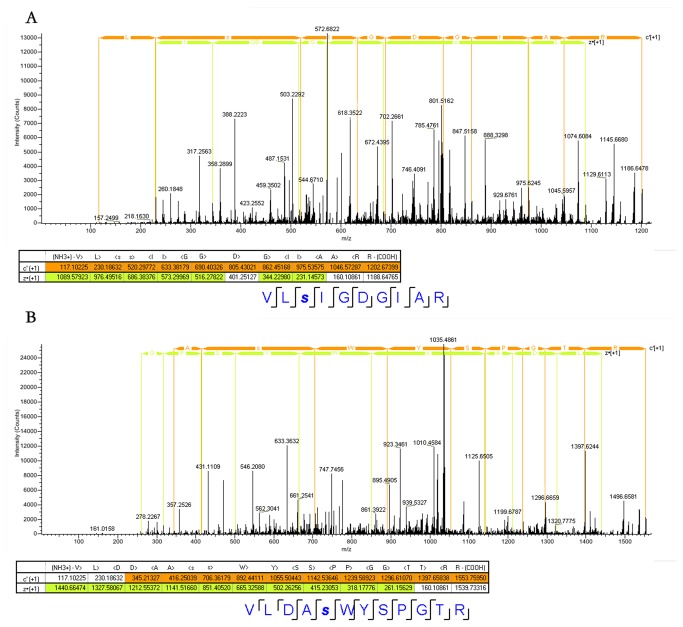
Nano-LC-ETD/MS/MS mass spectra of O-GlcNAcylated peptides. VL*S*IGDGIAR [(M+3H)^3+^ at m/z 401.89084] (A) from ATP synthase subunit alpha, and VLDA*S*WYSPGTR [(M+3H)^3+^ at m/z 518.92157] (B) from Thiosulfate sulfurtransferase.

In addition, ETD/MS/MS, which is the most trustworthy mass spectrometry tool to date for directly determination of the exact sites, was further used to verify the identified O-GlcNAcylation sites. The tryptic peptides from purified mitochondrial proteins were analyzed by directed mass spectrometry using Orbitrap Elite ETD. As a result, all the O-GlcNAcylated peptides were detected and verified. Figure 5 shows the ETD/MS/MS spectra of two O-GlcNAcylated peptides as examples. The c- and z- series of product ions clearly display a mass increase of 203.079 Da on Ser-76 directly indicating an O-GlcNAc on the Ser-76 of the identified O-GlcNAcylated peptide (VL***S***IGDGIAR) from ATP synthase subunit alpha (atp5A1) (Figure 5A). Figure 5B depicts the O-GlcNAc on the Ser-35 of the O-GlcNAcylated peptide (VLDA***S***WYSPGTR) from Thiosulfate sulfurtransferase (Tst). All other ETD/MS/MS spectra of the identified O-GlcNAcylated peptides were shown in supplemental figures (from Figure S1-Figure S11). Thus, so far the identified O-GlcNAcylated peptides and proteins have been verified successfully by the immunological method and the ETD/MS/MS analysis.

### 3.4: Data Analysis

O-GlcNAc is observed to be existent in rat liver mitochondria according to our western blot results (Figure 2). Totally 14 O-GlcNAcylation sites on 12 O-GlcNAcylated peptides, within 11 O-GlcNAcylated proteins are confidently identified by mass spectrometry. Using the Uniprot-designated subcellular annotation, we found that all of these identified O-GlcNAcylated proteins are located in mitochondria. Thus, this subcellular annotation together with a strict sampling method which was proven to produce high purity and integrity of mitochondrial fraction confirms the fact that the identified O-GlcNAcylated proteins are from mitochondrial fraction instead of other cellular components. It directly proves that O-GlcNAc modification definitely occurs in mitochondrial proteins. The molecular weight of the identified O-GlcNAcylated proteins is mostly between 34 kDa and 72 kDa, which is consistent with the western blot result (Figure 2A). Moreover, all of the 14 identified O-GlcNAcylation sites, which hitherto are undocumented in Swiss-Prot database (version of November 2012), are first confirmed in this study.

**Figure 6:**
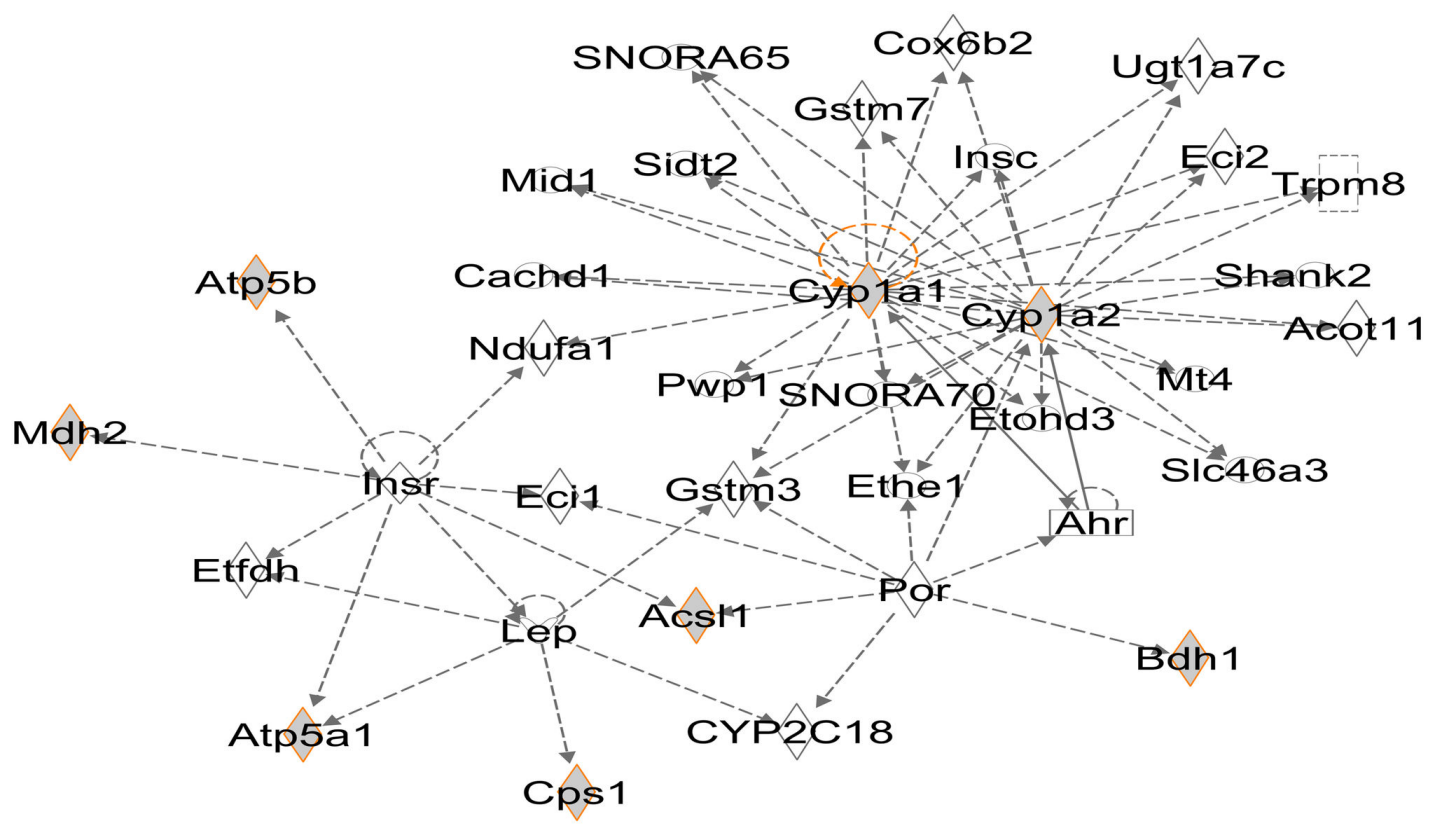
The most enriched sub-networks in IPA results. Gray symbols with orange rim are O-GlcNAcylated mitochondrial proteins identified in this study. Symbol ◊ is used for enzyme, □ for nuclear receptor, rectangle for ion channel, and ○ for others.

Notably, all of the 11 identified O-GlcNAcylated mitochondrial proteins are enzymes involved in a wide variety of biological processes. In fact, some of them, such as carbamoyl-phosphate synthase (Cps1), malate dehydrogenase (Mdh2) and long-chain-fatty-acid-CoA ligase (Acsl1) are very important intermediate metabolic enzymes involved in tricarboxylic acid (TCA) cycle, urea cycle, fatty acid metabolism and lipid metabolism. The interaction between these O-GlcNAcylated proteins was analyzed by IPA. Figure 6 shows the most enriched connectivity network. Interestingly, the 8 O-GlcNAcylated proteins out of the total 11 identified O-GlcNAcylated mitochondrial proteins are enriched in one sub-network. Two O-GlcNAcylated proteins, cytochrome P450 1A1 (Cyp1a1) and cytochrome P450 1A2 (Cyp1a2), consist of the center of this sub-network. Despite the lack of functional analysis of specific proteins, above analysis results still reveal that these identified O-GlcNAcylated mitochondrial proteins play an important role in organisms, and the O-GlcNAcylation may also be a significant factor in biological processes, which indicate that the effects of the O-GlcNAcylation need to be explored in mitochondria.

Furthermore, the extensive crosstalk between O-GlcNAc and O-phosphate is actively explored. Many studies have shown that the crosstalk can be more complicated than initially thought. Examples include competition or alternation between two modifications on the same site or adjacent sites [50-55], as well as reciprocal effect on proteins function by the adjacent occupancy of each modification [56]. Moreover, cells sometimes need to get a right balance between O-GlcNAcylation and O-phosphorylation for appropriate survival decisions [57]. Interestingly, in this study, among the 14 identified O-GlcNAcylation sites, 3 O-GlcNAcylation sites (Ser-537 on VLGTS#VESIMATEDR from Cps1, Ser-76 on VLS#IGDGIAR from Atp5a1, Thr-193 on KIFRECDHS#QT#D from Plcd1) were also annotated as phosphorylation sites at PhosphoSite Plus^®^ [58] (Figure S12A), which indicates a site-specific interplay between O-phosphorylation and O-GlcNAcylation on these sites, and indicates that this kind of interplay also exist in rat liver mitochondria as that in cytosol and nucleus. Moreover, analysis of the 11 identified O-GlcNAcylated proteins in PhosphoSite Plus^®^ revealed that, except for the 3 unrecorded proteins, the other 8 O-GlcNAcylated proteins are all phosphorylated proteins, which means competitive and alternate occupancy may occur at adjacent sites between the two modifications in mitochondria. It also hints that almost all O-GlcNAcylated proteins can be phosphorylated in mitochondria, which is the same situation with that in nucleolus and cytosol. Furthermore, the 8 O-GlcNAcylated proteins annotated as phosphorylated proteins in PhosphoSite Plus^®^ are known to be tyrosine phosphorylated. Among them, two O-GlcNAcylation sites (Ser-537 on Cps1, Ser-35 on Tst) are very near to the tyrosine phosphorylation sites (Tyr-529 on Cps1, Try-37 on Tst) (Figure S12B). The results substantiate the hypothesis of the interaction between tyrosine phosphorylation and O-GlcNAc modification [24], and suggest that this kind of interplay may also exist in mitochondria.

## Conclusions

In summary, for the first time, in highly purified mitochondrial proteins of rat liver, we not only discovered but also confirmed a number of O-GlcNAcylation sites by combination of CID/ETD mass spectrometry and immunoblot methods, which provided a direct and unambiguous evidence for O-GlcNAcylation sites mapping in mitochondria. And this study is also expected to arouse more attention to O-GlcNAcylation of mitochondrial proteins or proteins with other subcellular locations besides cytosol and nuclear, taking the study performed by Alfaro etc. for an example [40]. In addition, “cross talk” relationship between O-GlcNAcylation and phosphorylation was analyzed and implied to occur in mitochondria, which is worth studying in future. Furthermore, all of the new-found O-GlcNAcylated mitochondrial proteins are enzymes involved in a wide variety of biological processes and many important pathways. Although much remains unknown about the function of O-GlcNAcylation and the molecular mechanism of the interplay between O-GlcNAc and O-phosphate in mitochondria, our results indicate a previously unrecognized and potentially significant role of O-GlcNAcylation in mitochondria and will arouse more detailed studies.

## Supporting Information

Figure S1 **Nano-LC-ETD/MS/MS mass spectrum of O-GlcNAcylated peptide MA*S*TGEVACFGEGIHTAFLK [(M+3H)^3+^ at m/z 777.03754] from Carbamoyl-phosphate synthase.**
(TIF)

Figure S2 **Nano-LC-ETD/MS/MS mass spectrum of O-GlcNAcylated peptide MAS*T*GEVACFGEGIHTAFLK [(M+2H)^2+^ at m/z 1165.05164] from Carbamoyl-phosphate synthase.**
(TIF)

Figure S3 **Nano-LC-ETD/MS/MS mass spectrum of O-GlcNAcylated peptide VLGT*S*VESIMATEDR [(M+3H)^3+^ at m/z 604.29865] from Carbamoyl-phosphate synthase.**
(TIF)

Figure S4 **Nano-LC-ETD/MS/MS mass spectrum of O-GlcNAcylated peptide FGVEAF*S*DCLR [(M+3H)^3+^ at m/z 501.89545] from D-beta-hydroxybutyrate dehydrogenase.**
(TIF)

Figure S5 **Nano-LC-ETD/MS/MS mass spectrum of O-GlcNAcylated peptide VAVLGA*S*GGIGQPLSLLLK [(M+2H)^2+^ at m/z 998.59387] from Malate dehydrogenase.**
(TIF)

Figure S6 **Nano-LC-ETD/MS/MS mass spectrum of O-GlcNAcylated peptide LVLEVAQHLGE*S*TVR [(M+3H)^3+^ at m/z 618.66718] from ATP synthase subunit beta.**
(TIF)

Figure S7 **Nano-LC-ETD/MS/MS mass spectrum of O-GlcNAcylated peptide KIFRECDH*S*QTD [(M+4H)^4+^ at m/z 421.19293] from 1-phosphatidylinositol-4,5-bisphosphate phosphodiesterase delta-1.**
(TIF)

Figure S8 **Nano-LC-ETD/MS/MS mass spectrum of O-GlcNAcylated peptide KIFRECDHSQ*T*D [(M+3H)^3+^ at m/z 561.25507] from 1-phosphatidylinositol-4,5-bisphosphate phosphodiesterase delta-1.**
(TIF)

Figure S9 **Nano-LC-ETD/MS/MS mass spectrum of O-GlcNAcylated peptide QVAEMAECIG*S*ALIQK [(M+3H)^3+^ at m/z 650.98859] from Long-chain-fatty-acid--CoA ligase 1.**
(TIF)

Figure S10 **Nano-LC-ETD/MS/MS mass spectrum of O-GlcNAcylated peptide IT*S*CDIEVQDFVIPK [(M+3H)^3+^ at m/z 637.32123] from Cytochrome P450 2D10.**
(TIF)

Figure S11 **Nano-LC-ETD/MS/MS mass spectrum of O-GlcNAcylated peptide L*S*QQYGDVLQIR [(M+4H)^4+^ at m/z 406.46674] from Cytochrome P450 1A1 and 1A2.**
(TIF)

Figure S12 **The crosstalk between O-GlcNAc and O-phosphate.** Three identified O-GlcNAcylation sites were also annotated as phosphorylation sites at PhosphoSite Plus^®^ (A). Two identified O-GlcNAcylation sites were very near to the tyrosine phosphorylation sites (B) (blue). O-GlcNAc; (yellow): O-Phosphate; the pitch black characters are the identified peptide sequence.
(TIF)
