# Supplementary figures and images for: Discovery and Confirmation of O-GlcNAcylated Proteins in Rat Liver Mitochondria by Combination of Mass Spectrometry and Immunological Methods
Source: PLoS One. 2013 Oct 2;8(10):e76399. doi: 10.1371/journal.pone.0076399 (PMC3788734; doi:10.1371/journal.pone.0076399)

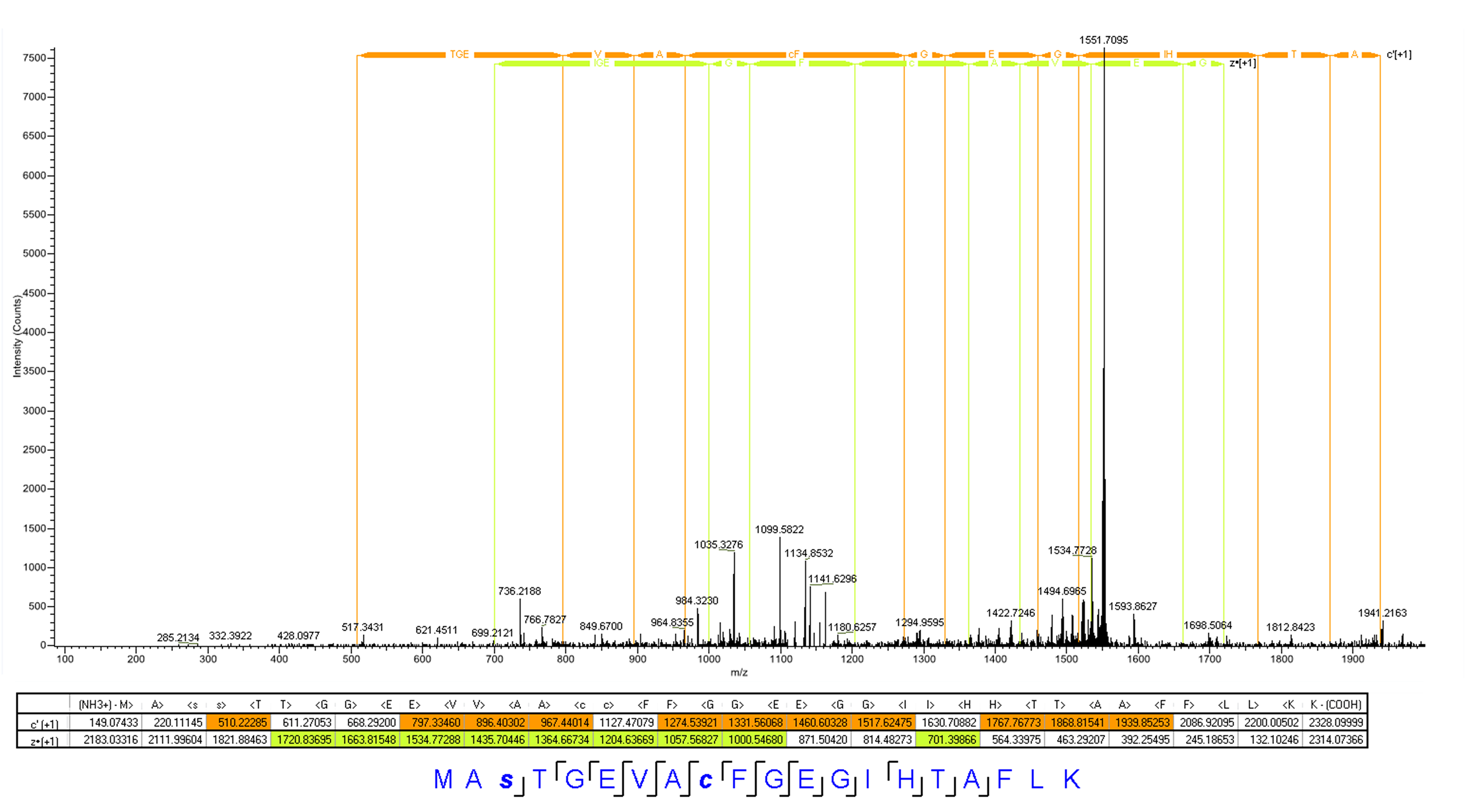

Supplement: Figure S1 — Nano-LC-ETD/MS/MS mass spectrum of O-GlcNAcylated peptide MASTGEVACFGEGIHTAFLK [(M+3H)3+ at m/z 777.03754] from Carbamoyl-phosphate synthase. (TIF) [file pone.0076399.s001.tif]

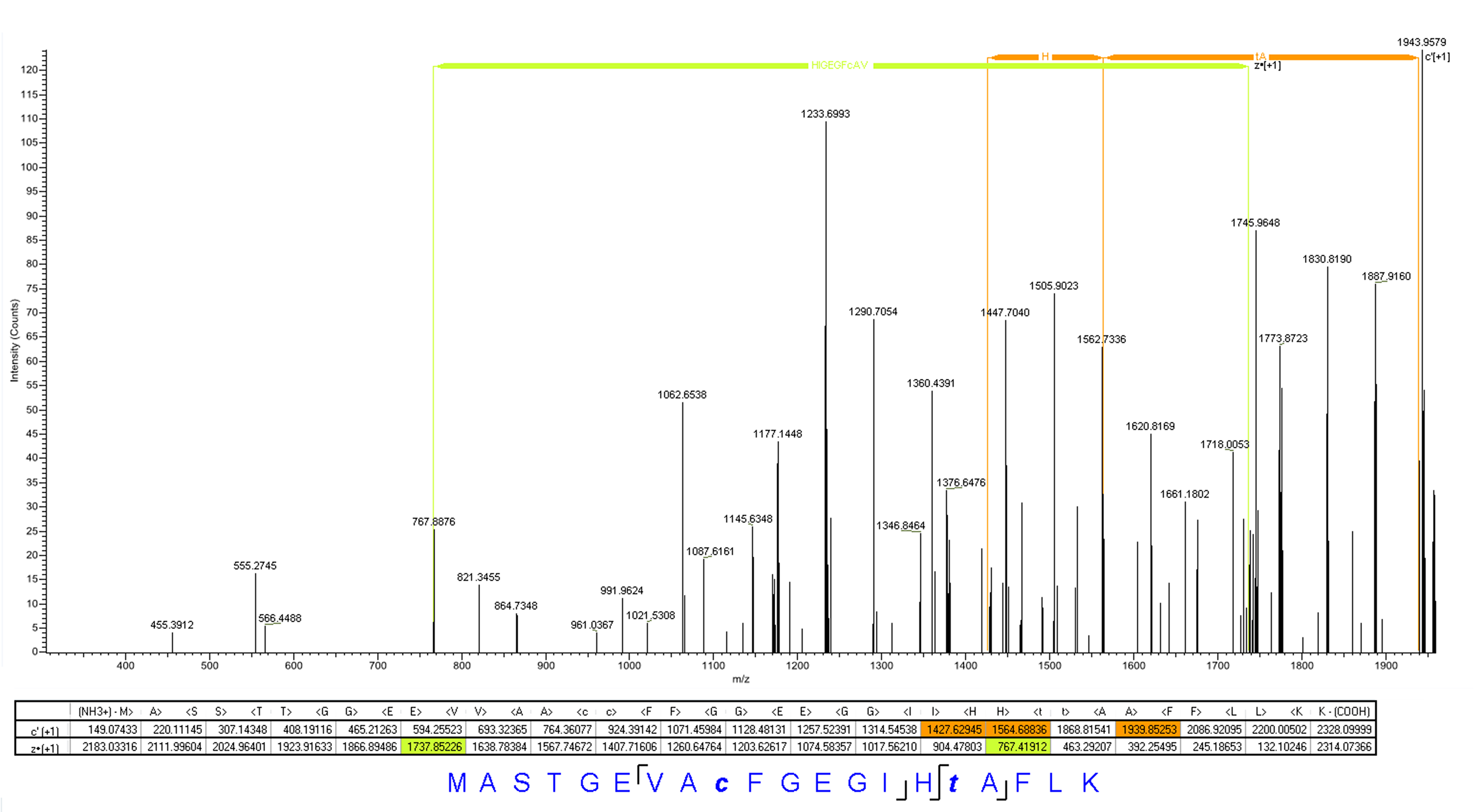

Supplement: Figure S2 — Nano-LC-ETD/MS/MS mass spectrum of O-GlcNAcylated peptide MASTGEVACFGEGIHTAFLK [(M+2H)2+ at m/z 1165.05164] from Carbamoyl-phosphate synthase. (TIF) [file pone.0076399.s002.tif]

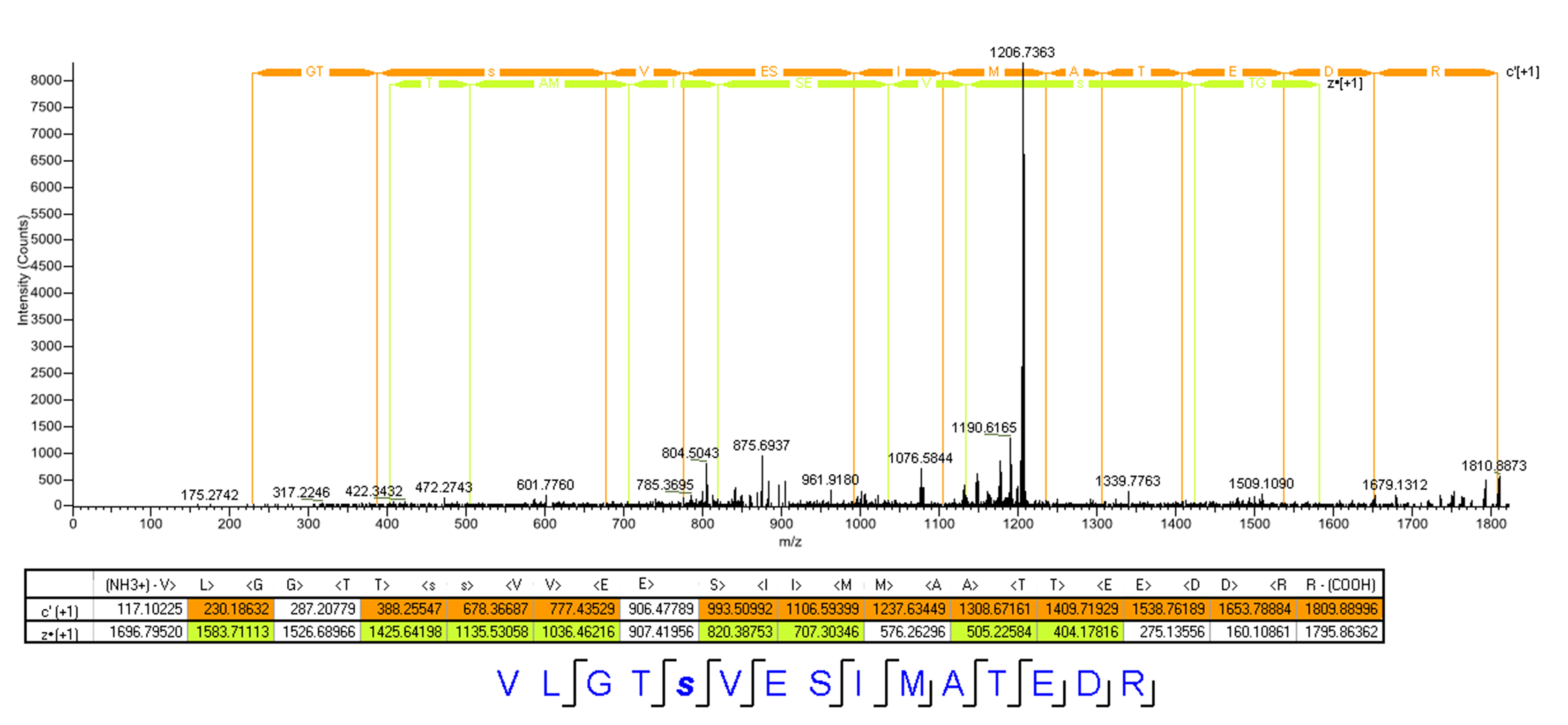

Supplement: Figure S3 — Nano-LC-ETD/MS/MS mass spectrum of O-GlcNAcylated peptide VLGTSVESIMATEDR [(M+3H)3+ at m/z 604.29865] from Carbamoyl-phosphate synthase. (TIF) [file pone.0076399.s003.tif]

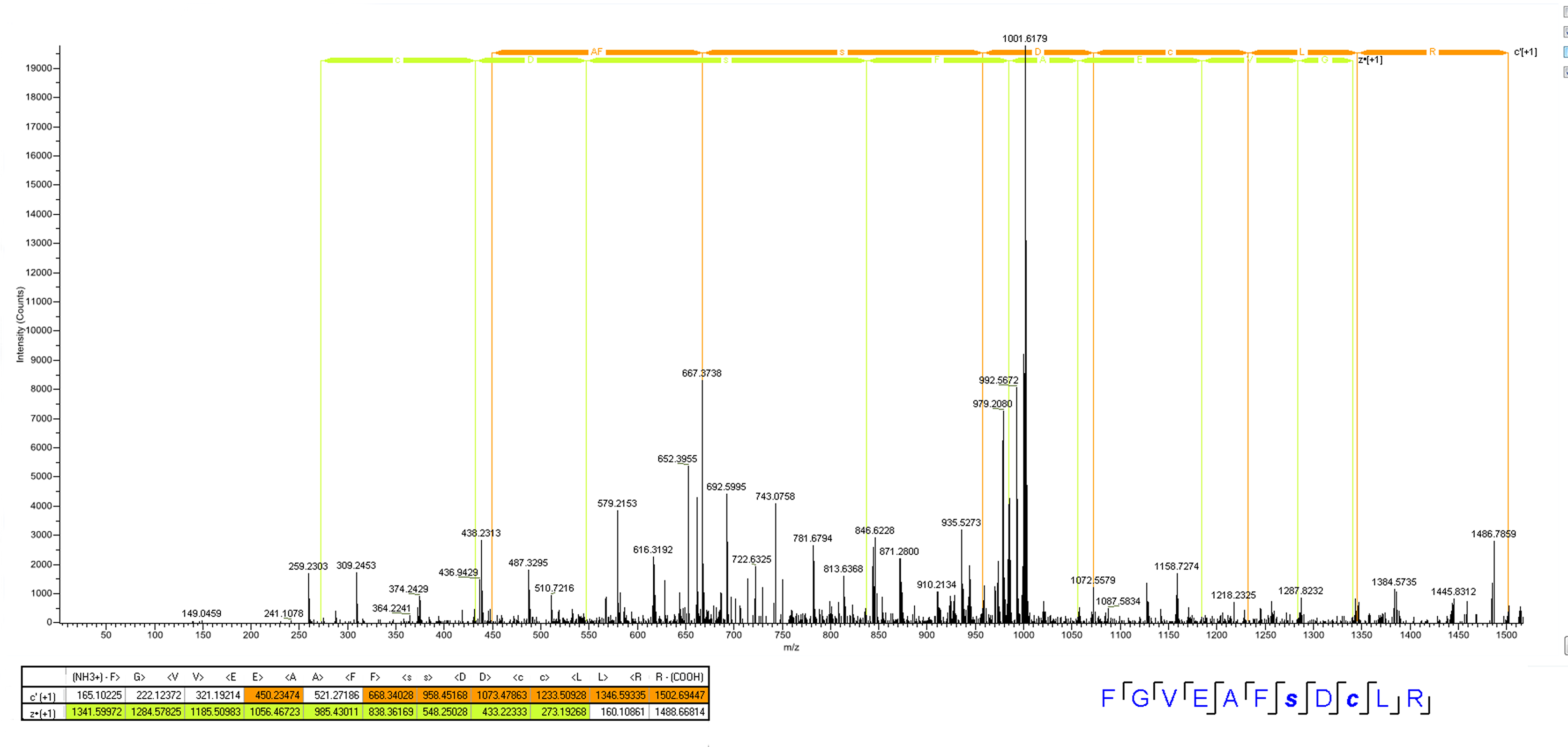

Supplement: Figure S4 — Nano-LC-ETD/MS/MS mass spectrum of O-GlcNAcylated peptide FGVEAFSDCLR [(M+3H)3+ at m/z 501.89545] from D-beta-hydroxybutyrate dehydrogenase. (TIF) [file pone.0076399.s004.tif]

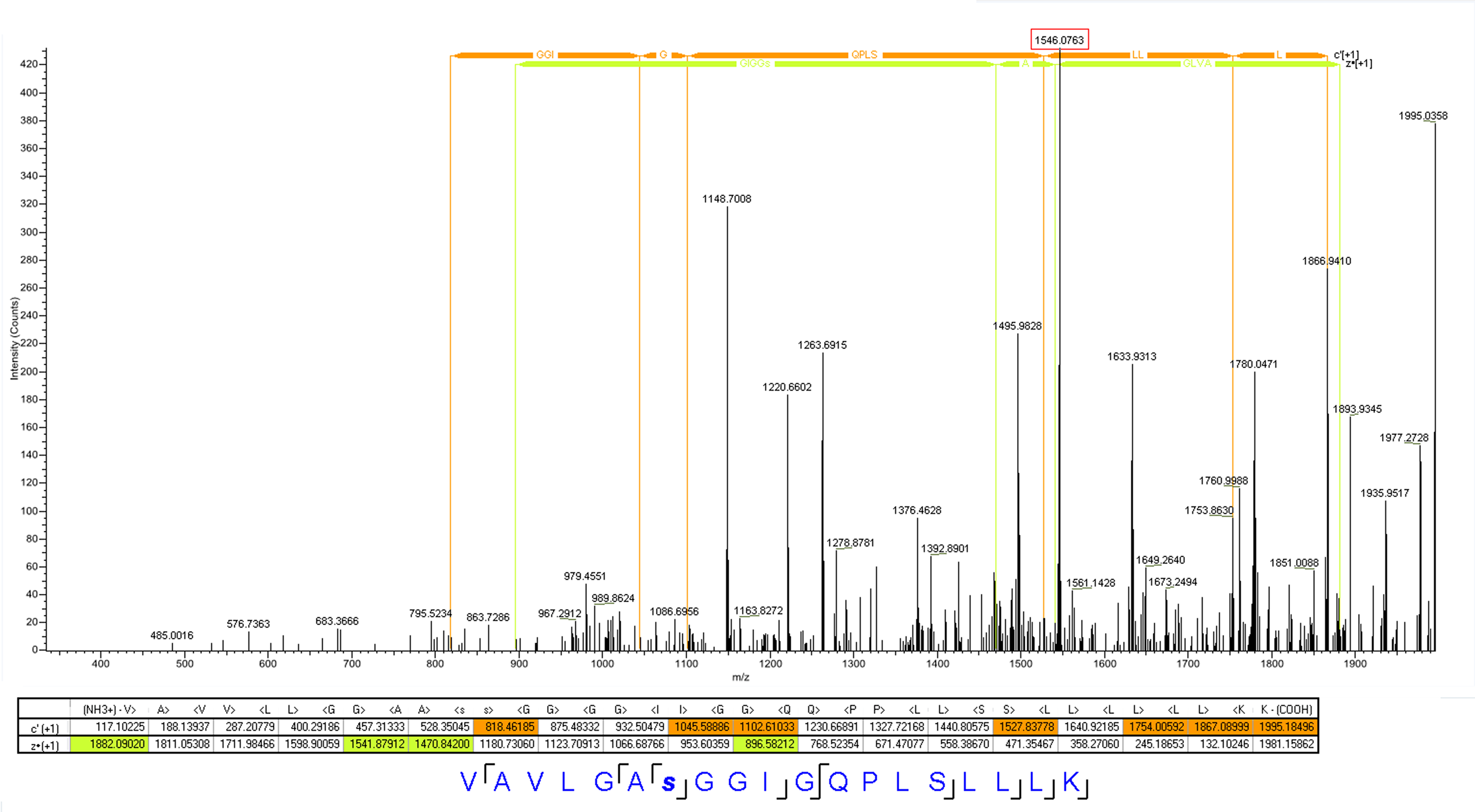

Supplement: Figure S5 — Nano-LC-ETD/MS/MS mass spectrum of O-GlcNAcylated peptide VAVLGASGGIGQPLSLLLK [(M+2H)2+ at m/z 998.59387] from Malate dehydrogenase. (TIF) [file pone.0076399.s005.tif]

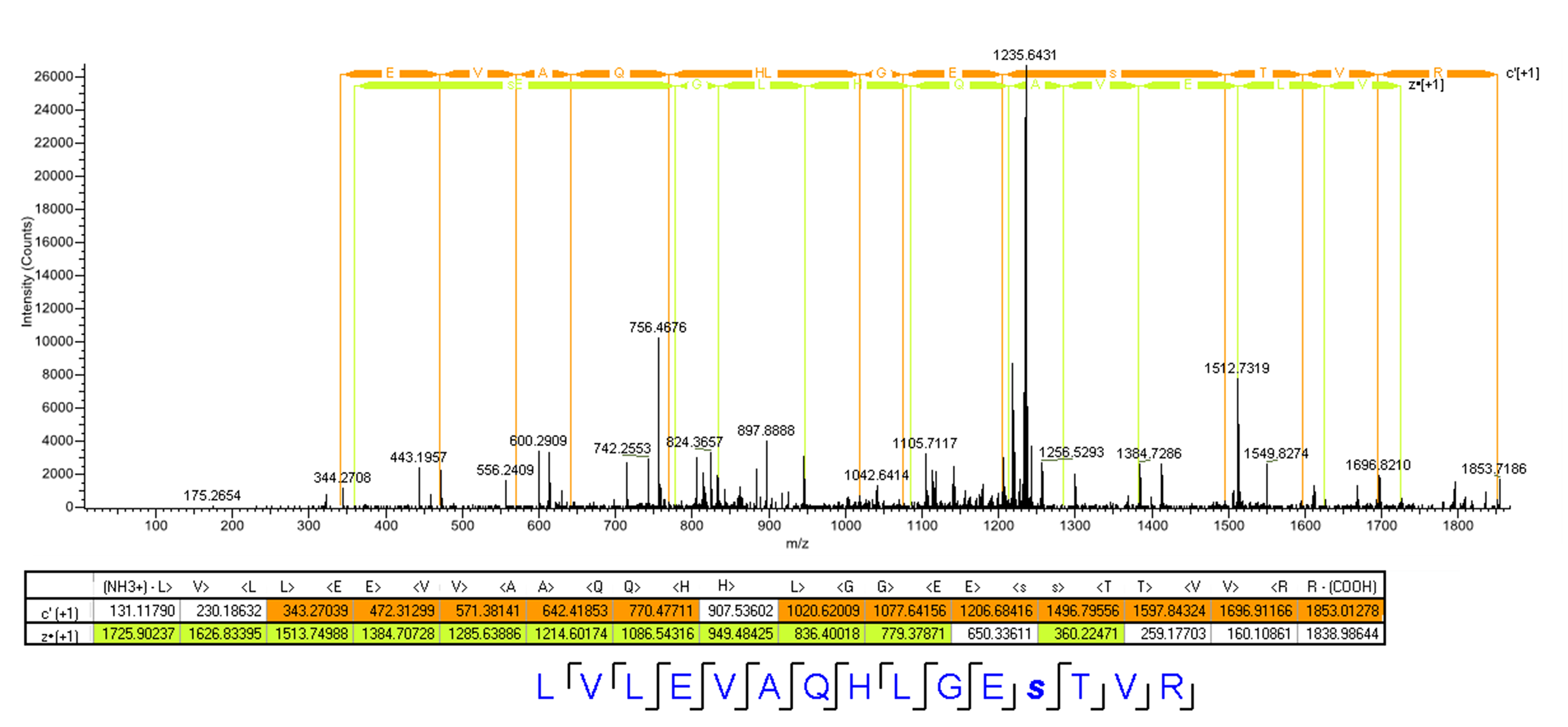

Supplement: Figure S6 — Nano-LC-ETD/MS/MS mass spectrum of O-GlcNAcylated peptide LVLEVAQHLGESTVR [(M+3H)3+ at m/z 618.66718] from ATP synthase subunit beta. (TIF) [file pone.0076399.s006.tif]

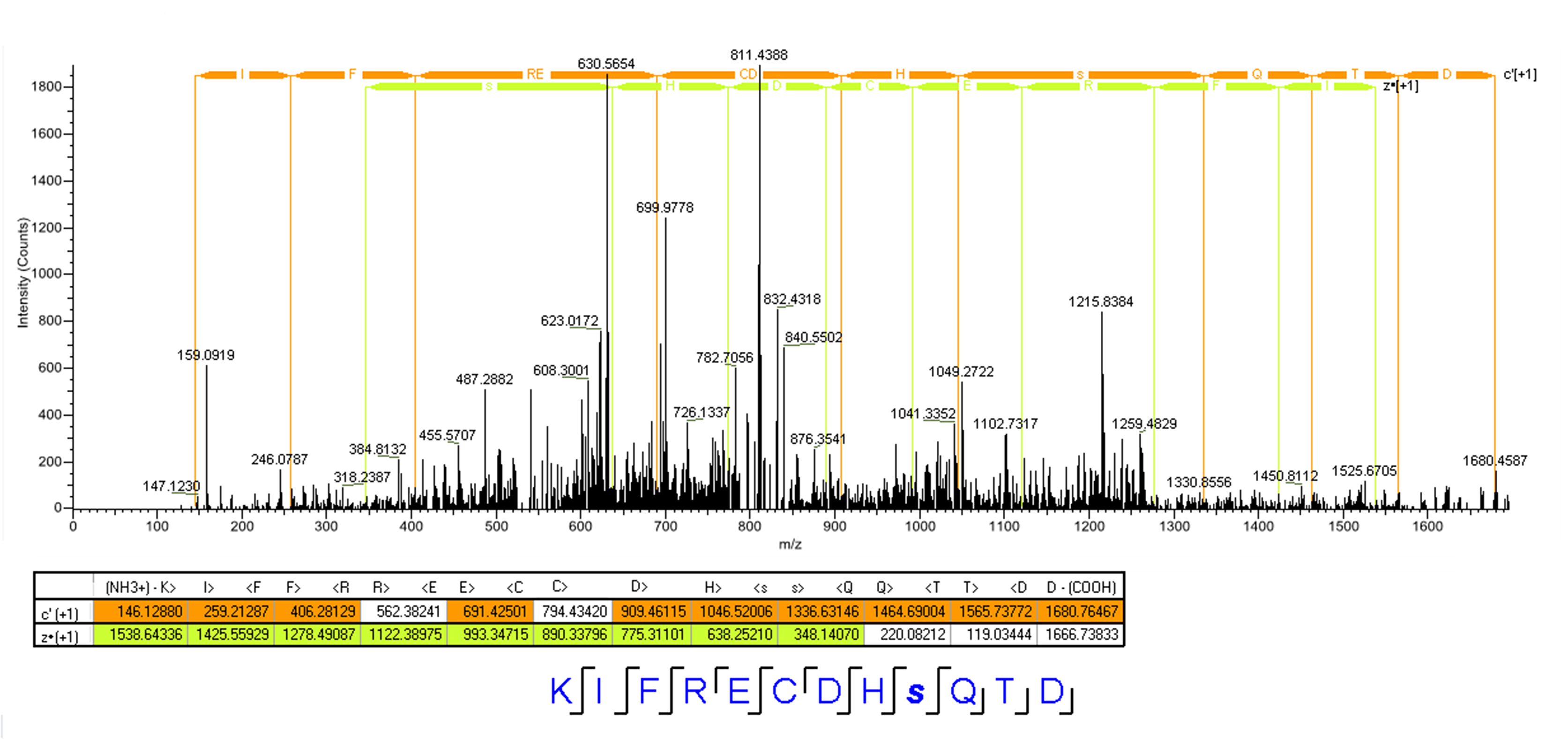

Supplement: Figure S7 — Nano-LC-ETD/MS/MS mass spectrum of O-GlcNAcylated peptide KIFRECDHSQTD [(M+4H)4+ at m/z 421.19293] from 1-phosphatidylinositol-4,5-bisphosphate phosphodiesterase delta-1. (TIF) [file pone.0076399.s007.tif]

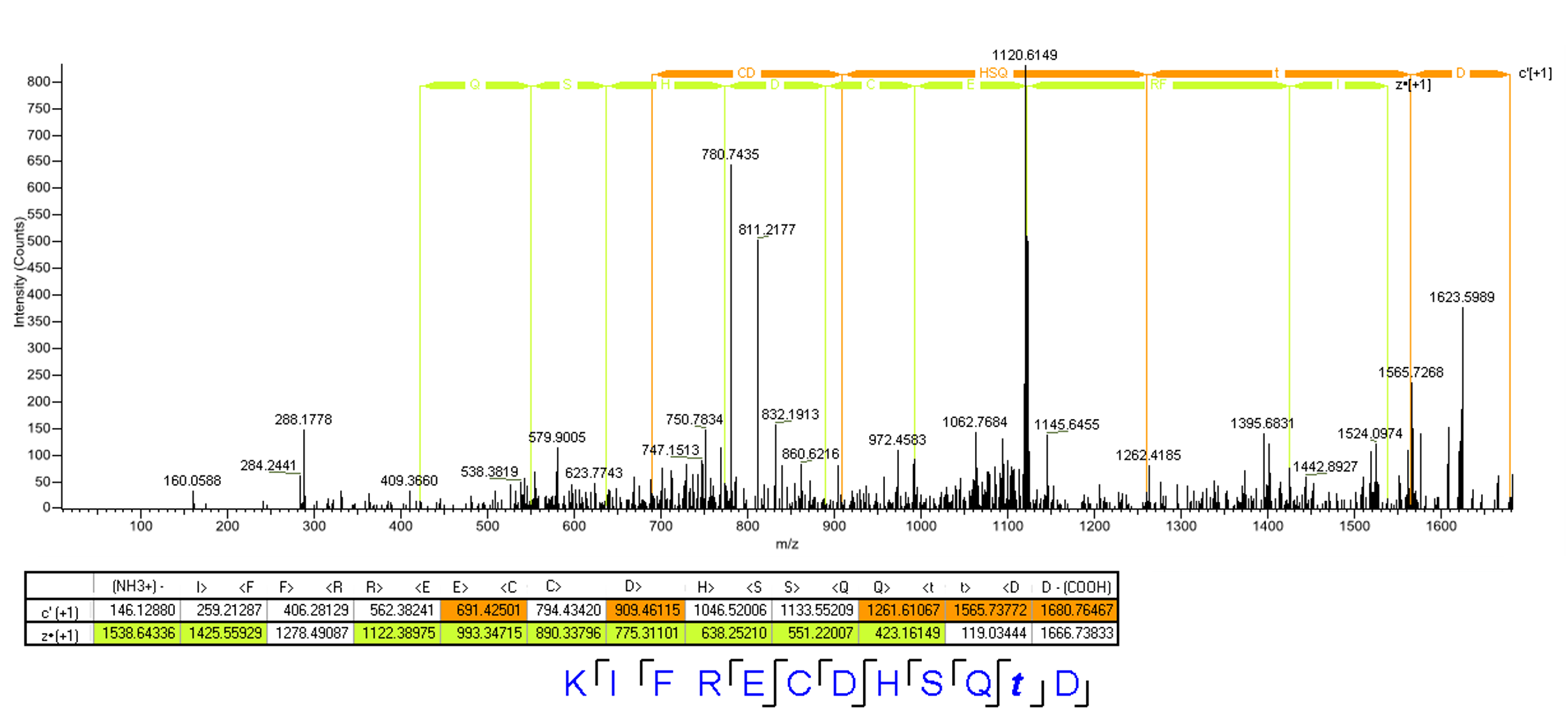

Supplement: Figure S8 — Nano-LC-ETD/MS/MS mass spectrum of O-GlcNAcylated peptide KIFRECDHSQTD [(M+3H)3+ at m/z 561.25507] from 1-phosphatidylinositol-4,5-bisphosphate phosphodiesterase delta-1. (TIF) [file pone.0076399.s008.tif]

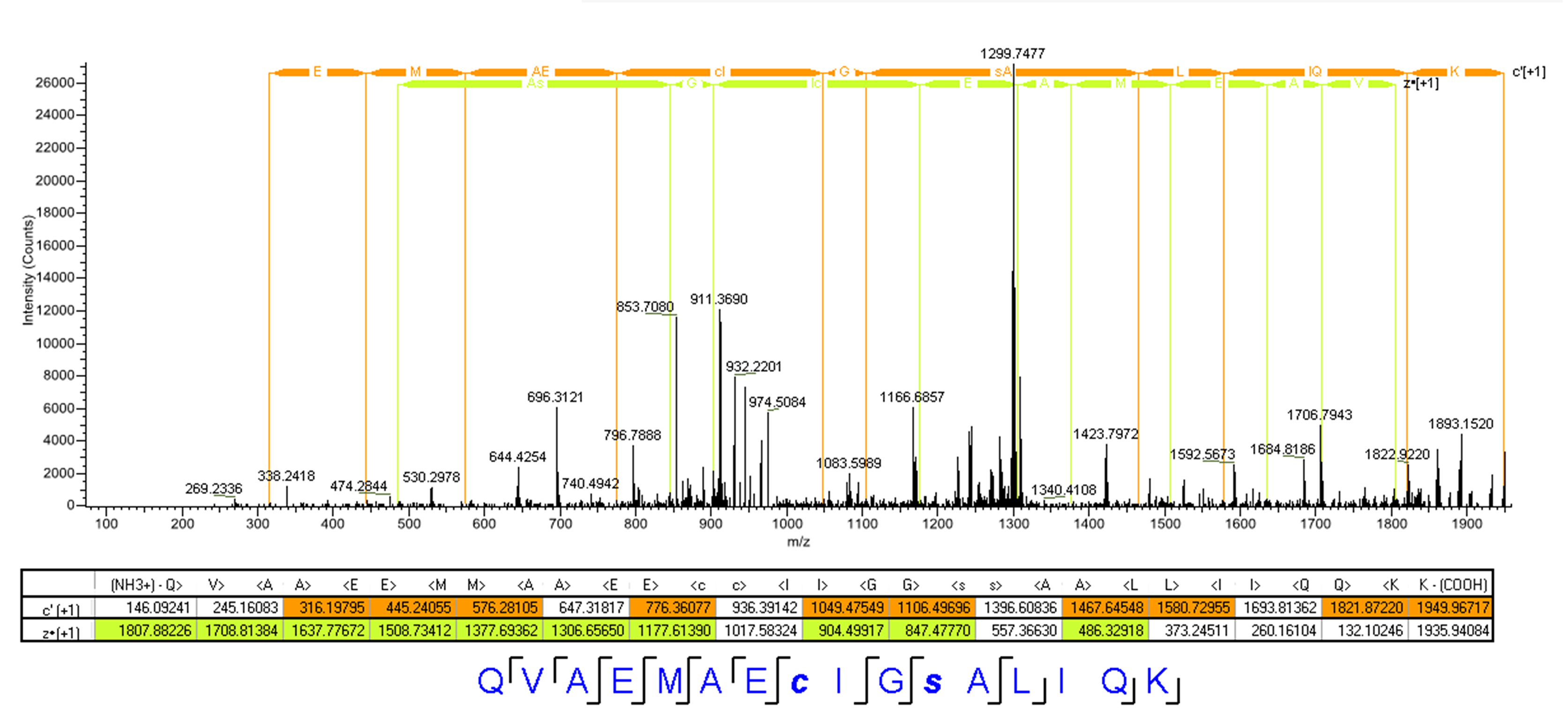

Supplement: Figure S9 — Nano-LC-ETD/MS/MS mass spectrum of O-GlcNAcylated peptide QVAEMAECIGSALIQK [(M+3H)3+ at m/z 650.98859] from Long-chain-fatty-acid--CoA ligase 1. (TIF) [file pone.0076399.s009.tif]

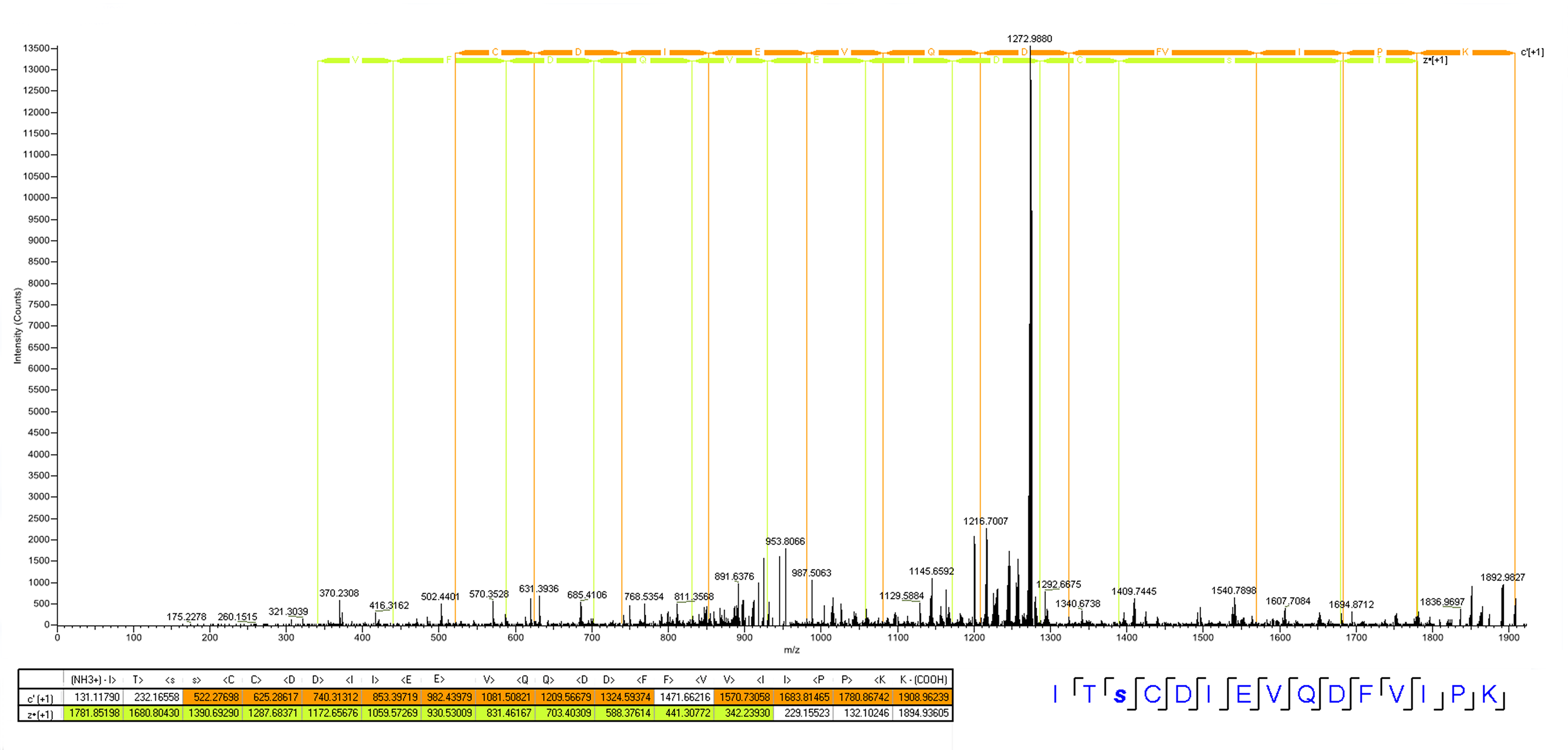

Supplement: Figure S10 — Nano-LC-ETD/MS/MS mass spectrum of O-GlcNAcylated peptide ITSCDIEVQDFVIPK [(M+3H)3+ at m/z 637.32123] from Cytochrome P450 2D10. (TIF) [file pone.0076399.s010.tif]

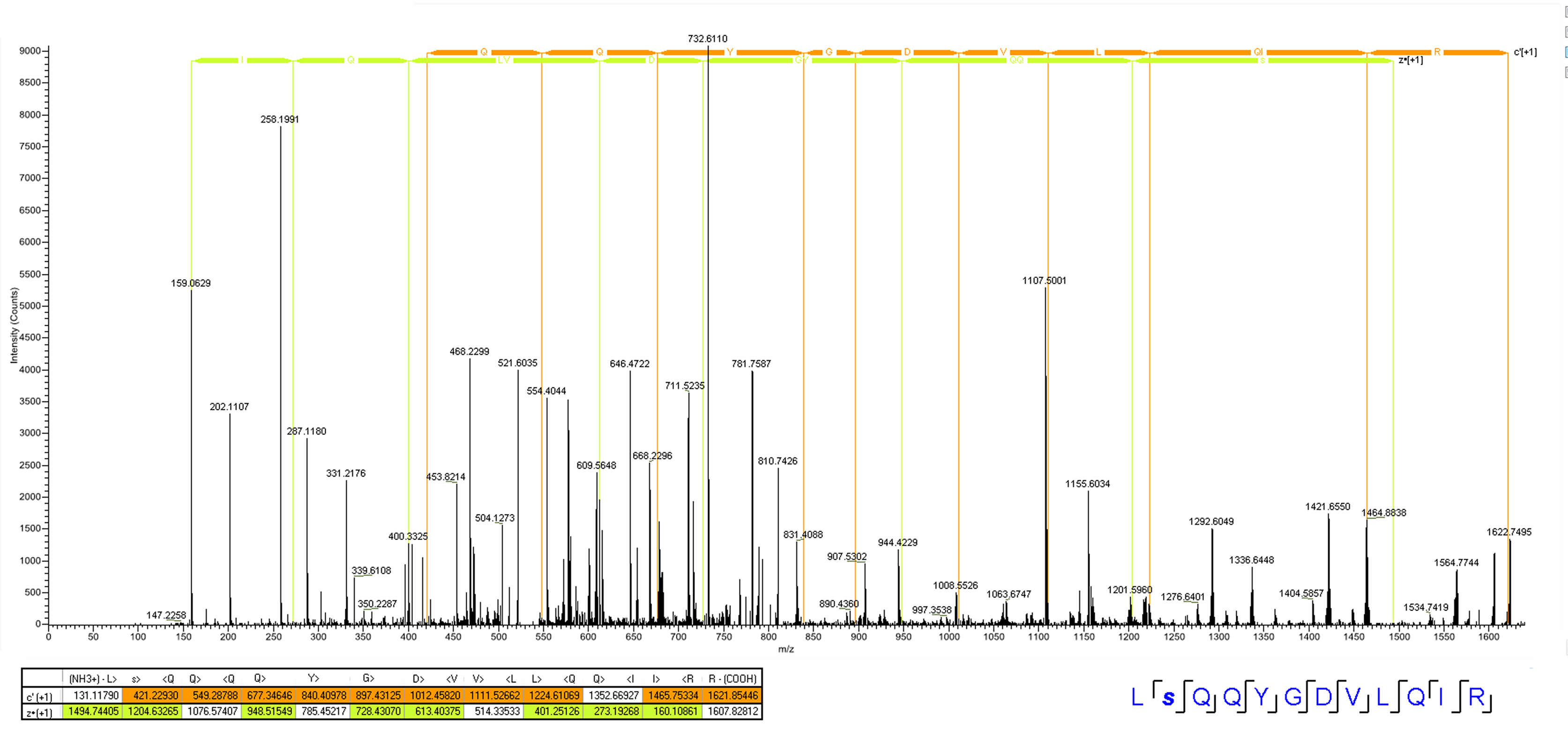

Supplement: Figure S11 — Nano-LC-ETD/MS/MS mass spectrum of O-GlcNAcylated peptide LSQQYGDVLQIR [(M+4H)4+ at m/z 406.46674] from Cytochrome P450 1A1 and 1A2. (TIF) [file pone.0076399.s011.tif]

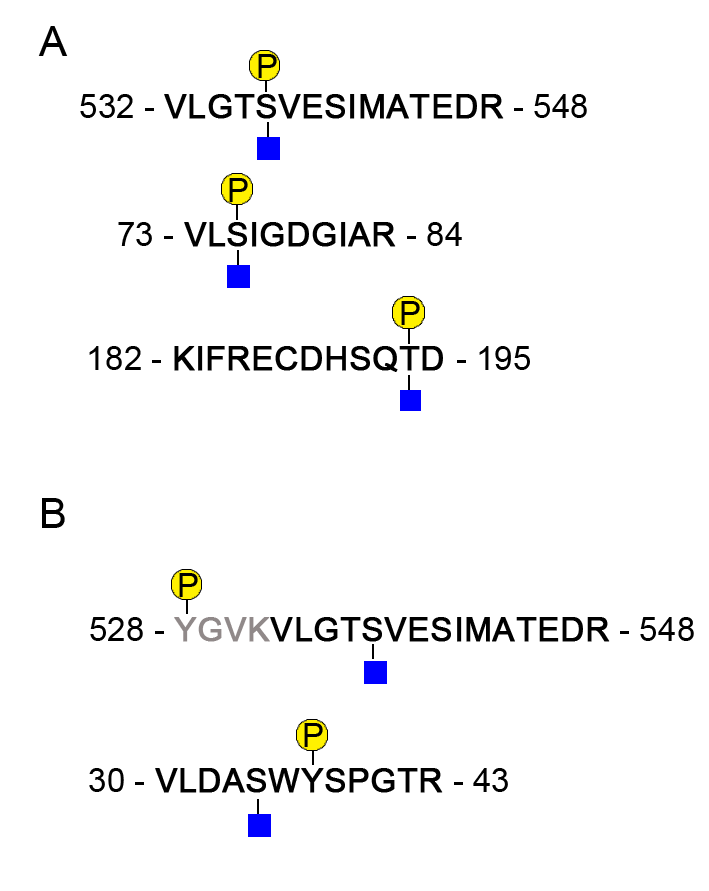

Supplement: Figure S12 — The crosstalk between O-GlcNAc and O-phosphate. Three identified O-GlcNAcylation sites were also annotated as phosphorylation sites at PhosphoSite Plus® (A). Two identified O-GlcNAcylation sites were very near to the tyrosine phosphorylation sites (B) (blue). O-GlcNAc; (yellow): O-Phosphate; the pitch black characters are the identified peptide sequence. (TIF) [file pone.0076399.s012.tif]
